# Quality of public school toilets and the frequency of changing sanitary napkins among students in public secondary schools in the City of Manila, Philippines

**DOI:** 10.1186/s41182-018-0131-8

**Published:** 2019-01-11

**Authors:** Chikako Katsuno, Ernesto R. Gregorio, Marian Fe Theresa C. Lomboy, Daisuke Nonaka, Paul Michael R. Hernandez, Crystal Amiel M. Estrada, Jennel Mae T. Pimentel, Rhea Marie Grace C. Bernadas, Jun Kobayashi

**Affiliations:** 1 Department of Global Health, Graduate School of Health Sciences, University of the Ryukyus, Uehara 207, Nishihara-cho, Okinawa, 903-0215 Japan; 2 Department of Health Promotion and Education, College of Public Health, University of the Philippines Manila/SEAMEO Regional Center for Public Health, Hospital Administration, Environmental & Occupational Health, 625 Pedro Gil Street, Ermita, Manila, Philippines; 3 Department of Environmental and Occupational Health, College of Public Health, University of the Philippines Manila/SEAMEO Regional Center for Public Health, Hospital Administration, Environmental & Occupational Health, 625 Pedro Gil Street, Ermita, Manila, Philippines

**Keywords:** Menstrual hygiene management, School health, Adolescents, Theory of planned behavior, The Philippines

## Abstract

**Background:**

In sub-tropical countries, poor menstrual hygiene management has been reported. One cause of poor menstrual hygiene management can be poor quality toilets. However, associations between poor quality toilets and menstrual-related behaviors have been poorly understood. The present study aimed to assess the association between the quality of school toilets and the frequency of changing sanitary napkins in school toilet among Filipino students.

**Methods:**

A cross-sectional study was conducted in six secondary schools of the City of Manila, Philippines, in 2017. A self-administered survey questionnaire with female students collected data on the outcome variable, self-reported daily frequency of changing sanitary napkins in school toilet, and other predictor variables. An observational survey collected data on the main predictor variable, surveyor-rated toilet quality variables. A total of 526 students were included in the analyses. Logistic regression with generalized estimating equation model was used to assess the association between the outcome and predictor variables.

**Results:**

No significant association was found both between toilet quality and the outcome. Although the association was not significant, the odds ratio (OR) of “sanitary bin is available in toilet” was 2.54 compared to “sanitary bin is not available in toilet.” The results of multivariate analysis showed that participants who reported stronger perceived behavioral control or stronger subjective norm were significantly more likely to change sanitary napkins, compared to those with lower perceived control score or lower subjective norm score, respectively (adjusted OR 2.29, 95% confidence interval 1.24 to 4.25; adjusted OR 2.63, 95% confidence interval 1.45 to 4.76).

**Conclusions:**

The present study showed that the quality of school toilets was not associated with the frequency of changing sanitary napkins among the studied population. However, it does not mean that the cause-effect relationship was rejected. Further studies involving more schools are necessary to confirm this relationship. Improving subjective norm and perceived behavior control might improve menstrual hygiene behavior.

## Background

For many years, menstruation and menstrual hygiene management have been overlooked in many countries all over the world. The global health community has reasonably focused its efforts on other health concerns like family planning, maternal health, and sex education, which are more directed toward addressing adolescent health needs [1]. In recent years, there is increasing recognition of the importance of promoting menstrual hygiene management in schools as it has been one of the agenda of a series of meetings on water, sanitation, and hygiene (WASH) in schools [2].

Menstruation is a natural part of the reproductive cycle, in which blood is lost through the vagina. However, in some countries, talking about menstruation remains a taboo. A review showed that in low- and middle-income countries, exclusion and shame still remains and it causes misconceptions and unhygienic practices during their period [3].

This study adopted the following definition of menstrual hygiene management (MHM) by the United Nations Children’s Fund (UNICEF) and the World Health Organization (WHO) which describes adequate MHM as a condition wherein:

Women and adolescent girls are using a clean menstrual management material to absorb or collect menstrual blood, that can be changed in privacy as often as necessary for the duration of a menstrual period, using soap and water for washing the body as required, and having access to facilities to dispose of used menstrual management materials [4].

Poor menstrual hygiene management can lead to a wide range of negative consequences including health, psychosocial, and educational consequences. Health consequences include urinary tract infection, menstrual pain, and anemia. Psychosocial consequences include fear, shame, teasing, bullying, self-exclusion, and less interaction. Educational consequences include school absenteeism [5], less concentration during class, and dropout [6]. Several studies have explained the impact of poor MHM on these outcomes. For example, a study reported that students who did not use sanitary napkins were significantly more likely to be absent from schools [7]. In another study, women who used reusable menstrual materials were significantly more likely to have symptoms of urogenital infection [8].

Inadequate water and sanitation facilities have been reported to impede MHM among students especially in low-income countries [2]. According to a study in India in 2012, reasons for school absenteeism among female students included the lack of proper disposal facility for sanitary napkins (75% of the respondents), lack of a continuous water supply (67.5%), abdominal pain and physical discomfort (65%), advice of grandmother (47%), fear of leakage (23.7%), and a feeling of being socially withdrawn during menstruation (5.4%) [9].

Although many tropical countries have been facing menstrual concerns and its consequences, we conducted the present study in the Philippines following a research collaboration program between the University of the Philippines Manila and University of the Ryukyus. In the Philippines, there are only few qualitative studies that assessed MHM and the experiences of students with regard to menstrual hygiene management. A qualitative case report published by UNICEF in 2012 described the challenges faced by female students related to MHM such as confusion about effective management practices, difficulty of managing menstruation, fear of leaks, stains, teasing, shame and embarrassment, menstrual odor, menstrual pain, discomfort, and fatigue. Moreover, some environmental and psychosocial determinants of MHM were likewise identified in the report. These included inadequate WASH facilities in schools, insufficient knowledge, practical guidance and support, and poor accessibility to preferred materials [10]. This qualitative study described the possible association between inadequate WASH facilities in schools and menstrual hygiene management practices. However, there were no quantitative studies done which confirmed this potential association. A study by Ellis et al. examined the WASH conditions that affect Filipino female students to hygienically manage their menstruation. In the study, qualitative data were collected from 13 schools in Metro Manila, Masbate, and South Central Mindanao. The study showed that the key barriers to effective menstrual management in Masbate and Metro Manila were unreliable access to water, lack of disposal mechanisms for menstrual materials, unclean facilities, and insufficient number of toilets [11].

Most of the studies done in the past examined the frequency of changing menstrual materials per day as a measure to describe a girl’s menstrual hygiene practices [12]. However, there were only three studies that examined frequency of changing menstrual materials in school. In India, one study revealed 20.6% of the girls changed menstrual materials at school or at college [13]. A study conducted in Ethiopia showed that only 14.5% of girls changed menstrual materials at school [7]. A study in Indonesia revealed that, based on in-depth interviews and focus group discussions (FGDs), students reported to never or rarely changed materials at school [14].

It is clear that more studies assessing menstrual hygiene management are required. As a consequence, the lack of evidence can further hinder the planning and implementation of programs which can effectively address the gaps and challenges in MHM in the country.

This study aimed to determine the association between the quality of school toilets and the self-reported daily frequency of changing sanitary napkins among grade eight female students in public secondary schools in the City of Manila.

## Methods

### Study site and population

This cross-sectional study was conducted in the City of Manila, the capital city of the Philippines. Its total population in 2016 was 1.78 million [15]. There were 33 public secondary schools located in the city with 84,213 students in the year 2015–2016; 43,147 (51.2%) are females [16].

The study employed a three-stage cluster sampling design—district, school, and students. In the first stage, two out of the six districts were selected based on simple random sampling. There are 12 schools located in the two districts. In the second stage, out of the 12 schools, 6 schools that met the following criteria were included: (1) availability of a toilet that can be continuously used by the grade eight female students, (2) the school has only one toilet that can be used by female grade eight students, and (3) the school has more than 100 female grade eight students. In the third stage, according to the difference in the size of schools and classes, we selected a minimum of 8 to maximum of 13 classes from each of the schools to include approximately 100 students. All female students who belonged to those classes were invited.

The target sample size was 600 as determined using power analysis. For multivariate analysis, each independent variable should have at least 10 participants with or without an outcome event as explained by [17]. Observing this rule, the number of independent variables (9) was multiplied by the minimum number of required participants (10). This resulted to 90. The prevalence used to compute for sample size was 20% [13]. Since 90 is only 20% of the population, the computed sample size should be multiplied by a factor of 5 to cover 100% of the population. This resulted to 450. Accounting for a non-response rate of 30%, the sample size was then identified to be 600 students.

Approximately 850 students were invited to participate in the study. Only 637 or 75% responded to the questionnaire. For the analyses, 51 students were excluded because they had not had menarche yet, 2 students were excluded because they did not use sanitary napkin to absorb blood, and 58 students were excluded because they did not respond completely. Finally, data from 526 students were analyzed.

### Data collection

Data were collected using a self-administered questionnaire and an observation checklist. A self-administered questionnaire was used to collect information from grade eight female students. The questionnaire was translated from English to Filipino and then back translated to English to check its face validity. After which, it was pre-tested among grade eight students in another district in Manila for clarity and cultural sensitivity. An observational survey was conducted by surveyors to assess toilet quality by using a checklist. The checklist was developed based on information gathered from the ASEAN Public Toilet Standard (2012) and UNICEF WASH in Schools Monitoring Package (2011) [18, 19].

### Variables and measurements

1. Frequency of changing sanitary napkins

The outcome of the present study was self-reported daily frequency of changing sanitary napkins in school toilets for the last menstrual period. The question item was “In your last menstrual period, how many times did you change sanitary napkins in school toilets in a day?” The response options to the question were “none,” “once,” “twice,” “three times,” or “four times or more.”

2. Toilet quality

Predictors of interest in the present study were both the perceived and the observed toilet quality. Regarding the perceived toilet quality, the perceptions of grade eight female students on the quality of school toilets were gathered through a self-administered questionnaire. They were asked to rate the following: privacy, availability of sanitary bin, perceived toilet bowl cleanliness, perceived smell of the toilet, and availability of hand wash facility using a 5-point Likert scale where 5 corresponds to strongly agree and 1 means strongly disagree. The responses were then categorized as follows: “agree,” “disagree,” and “neutral.” Observed toilet quality was determined though the following variables: “door is intact,” “door can be locked,” “toilet interior is not visible from the outside,” “availability of sanitary bin,” “toilet seat and bowl is clean,” and “taps are functional (at least half of the taps have water consistently flowing upon turning on).” The following variables “door is intact,” “door can be locked,” “toilet interior is not visible from the outside,” and “toilet seat and bowl is clean” were assessed as either good or bad. “Availability of sanitary bin” was assessed as follows: “sanitary bin is available in each cubicle,” “sanitary bin is available in the toilet,” and “sanitary bin is not available in the toilet.” The assessment was done twice (the first day and the second day) by two surveyors independently (principal investigator and a female research assistant) and without prior notification to the schools to prevent them from cleaning the toilet prior to observations. The inter-rater reliability and the intra-rater reliability were computed by using Cohen’s kappa. Only the parameters with kappa values showing substantial agreement (kappa values of 0.61 to 1.00) [20] in both inter-rater and intra-rater reliability were used as predictor variables. The obtained variables were as follows: “door is intact,” “door can be locked,” “toilet interior is not visible from the outside,” “observed availability of sanitary bin,” “observed toilet bowl cleanliness,” and “taps are functional (at least half of the taps are functional).”

3. Theory of planned behavior variables

The variables of the theory of planned behavior (TPB) were developed based on the guidelines provided by Icek Ajzen and Jillian J Francis [21, 22]. The behavior studied was “changing sanitary napkins in school toilets.” The internal consistency of each variable was confirmed after the pre-test and also after the actual survey. The Cronbach’s alpha value of each variable obtained from the pre-test showed higher internal consistency. Therefore, all the variables that were used in the pre-test were obtained in the actual survey. However, after the actual survey, the Cronbach’s alpha for “attitude” and “perceived behavioral control” were 0.28 and 0.32, respectively. Therefore, only one question item was retained to measure attitude and perceived behavioral control. The retained question items and scoring system for each variable were described as follows. Attitude was measured using the question “changing sanitary napkins in school toilets is a good practice” with responses categorized into three levels: 1 = disagree to 3 = agree with higher scores indicating stronger health attitude. Perceived behavioral control was measured through the statement “For me to change sanitary napkins in school is easy” with responses categorized into three levels: 1 = disagree to 3 = agree with higher scores indicating greater perceived behavioral control. Subjective norm was assessed though following items: (1) It is expected of me that I change sanitary napkins in school toilets; (2) My family expects that I should change sanitary napkins in school toilets; and (3) My friends want me to change sanitary napkins in school toilets. The sum of the scores for the three items on subjective norm was obtained. The questions were rated as 1 = strongly disagree to 5 = strongly agree with higher scores indicating stronger perceived social pressure to change sanitary napkins. A total score was categorized into three levels: 3–9, 10–12, and more than 12, with higher scores indicating a stronger perceived social pressure to change sanitary napkins.

4. Socio-economic status

The father’s highest educational attainment was used as a surrogate measure of the student’s socio-economic status. In the absence of such information, the mother’s educational attainment was used instead. The response options were “primary,” “secondary,” and “tertiary” level of education.

5. Level of menstrual flow

The student’s level of menstrual flow in the past 3 months was asked [23]. The responses were categorized into two: “light or moderate” and “heavy.”

6. Other data

Other relevant data were gathered. These included the student’s age and age of menarche.

### Conceptual framework

This study used two theoretical models. Firstly, we modified the ecological model used in the previous study by Haver et al. wherein they explored the impacts and potential risks of menstrual hygiene challenges among school girls in Masbate, Philippines [10]. In this framework, five factors were studied. Excluding societal factors, the ones used in the present study were biological, personal, interpersonal, and environmental factors. Secondly, the present study applied selected constructs from the theory of planned behavior (TPB) variables to assess the students’ perceptions toward changing sanitary napkins. TPB, as proposed by Icek Ajzen, predicts deliberate behavior. This theory suggests that one’s behavior is determined by one’s intention to perform certain behavior, and the intention is determined by the following: attitude toward behavior, subjective norm, and perceived behavioral control (PBC). The theory has been used in several studies for explaining health behavior among adolescents such as in dieting [24] and condom use [25].

In the present study, biological factors included the level of menstrual flow; individual factors included father’s education, attitude, and perceived behavioral control; interpersonal factors included subjective norms; and the environmental factors included toilet quality.

### Data analysis

SPSS version 24 was used for descriptive analyses and Cohen’s kappa statistics computation. For the multivariate analysis to assess the association between each predictor variable of interest and self-reported daily frequency of changing sanitary napkins, logistic regression with generalized estimating equation (GEE) model was conducted by using STATA 14. The outcome was dichotomized into “changed” or “not changed.” Of the six predictor variables on observed toilet quality, only two were eventually included in the multivariate analysis to determine association, since multicollinearity was a concern. These variables were also easier for the schools to address. Exchangeable working correlation matrix was applied in the GEE model. Model-based estimate was applied for variance-covariance matrix because the estimate method was appropriate for the data set where the number of the cluster was small [26, 27].

## Results

### Characteristics of participants

The median age of the study participants was 13 years old while the median age of menarche was 12 years old (Table 1). Forty-seven percent (*n* = 246) of the study participants reported their father’s educational attainment was secondary completion, followed by secondary incompletion (32%, *n* = 167) and tertiary completion (21%, *n* = 113). Almost all students (80%, *n* = 423) reported their level of menstrual flow as light to moderate. Twenty percent (*n* = 103) of the students reported a heavy level of menstrual flow.

**Table 1:** Socio-economic and menstrual hygiene characteristics of respondents (*n* = 526)

Characteristic	Number	Percentage
Age (years), median (IQR)	13	(13–14)
Father’s educational attainment		
Secondary incompletion	167	31.8
Secondary	246	46.7
Tertiary	113	21.5
Age of menarche (years), median (IQR)	12	(11–12)
Frequency of changing napkins in school toilets		
Once	239	45.4
Twice	124	23.6
Three times	37	7.0
Four times or more	3	0.6
Not changed	123	23.4
Daily frequency of changing napkins in any place		
Once	17	3.2
Twice	122	23.2
Three times	237	45.1
Four times or more	150	28.5
Not changed	0	0.0
Level of the menstrual flow		
Light	17	3.2
Moderate	406	77.2
Heavy	103	19.6

Most of the students (77%; *n* = 403) changed napkins at least once in school toilets whereas 23% (*n* = 123) of the study participants did not change sanitary napkins in school toilets. Most of the students reported changing sanitary napkins once, followed by twice and none. All students reported that they changed sanitary napkins at least once a day in either school toilets or somewhere else.

### Association with changing sanitary napkins in school toilets

Bivariate analysis showed that the toilet quality variables were not significantly associated with changing sanitary napkins in school toilets (Table 2). Consistent with the results of bivariate analysis, no toilet quality variables were found to be associated with changing sanitary napkins during the multivariate analysis (Table 3). However, there was a tendency for students who belong to schools with available sanitary bins either in the toilet room or in the cubicles to change their sanitary napkins in school toilets respectively (adjusted odds ratio 2.54, 95% confidence interval 0.76 to 8.40; AOR 2.19, 95% CI 0.84 to 5.74). Students’ age was significantly associated with changing sanitary napkins in school in the bivariate analysis (crude odds ratio 1.33; 95% CI 1.02 to 1.74). However, age became insignificant in the multivariate analysis (AOR 1.33; 95% CI 0.99 to 1.80). Bivariate analysis showed that the educational attainment of the father had no significant association with changing sanitary napkins. The multivariate analysis, on the other hand, showed that participants whose father’s educational attainment was at tertiary level were significantly more likely to change sanitary napkins (AOR 2.09; 95% CI 1.08 to 4.02), compared to participants whose father did not complete secondary education.

**Table 2:** Bivariate association with frequency of changing sanitary napkins in school toilets

Variables	Changed		Not changed		Odds ratio	95% CI	*P* value
	*n*	%	*n*	%			
Observed availability of sanitary bin							
Not available	58	65.2	31	34.8	1		
Available in toilet	62	83.8	12	16.2	2.04	0.59, 12.9	0.197
Available in cubicles	283	78.0	80	22.0	2.76	0.69, 6.00	0.197
Observed toilet bowl cleanliness*							
Bad	127	75.1	42	24.9	1		
Good	276	69.2	123	30.8	1.01	0.36, 2.80	0.990
Perceived privacy							
Disagree	41	73.2	15	26.8	1		
Neutral	45	75.0	15	25.0	1.09	0.47, 2.50	0.845
Agree	317	77.3	93	22.7	1.18	0.62, 2.25	0.612
Perceived availability of sanitary bin							
Disagree	40	67.8	19	32.2	1		
Neutral	36	81.8	8	18.2	2.04	0.77, 5.39	0.152
Agree	327	77.3	96	22.7	1.33	0.73, 2.44	0.357
Perceived toilet bowl cleanliness							
Disagree	73	79.3	19	20.7	1		
Neutral	53	75.7	17	24.3	0.82	0.39, 1.72	0.603
Agree	277	76.1	87	23.9	0.79	0.45, 1.40	0.416
Perceived smell							
Disagree	104	80.6	25	19.4	1		
Neutral	54	80.6	13	19.4	1.11	0.52, 2.35	0.790
Agree	245	74.2	85	25.8	0.75	0.46, 1.24	0.262
Perceived availability of hand wash facility							
Disagree	47	74.6	16	25.4	1		
Neutral	34	75.6	11	24.4	1.26	0.52, 3.07	0.605
Agree	322	77.0	96	23.0	1.26	0.68, 2.32	0.462
Age					1.33	1.02, 1.74	0.036
Father’s educational attainment							
Secondary incompletion	116	69.5	51	30.5	1		
Secondary	193	78.5	53	21.5	1.39	0.86, 2.21	0.162
Tertiary	94	83.2	19	16.8	1.75	0.96, 3.20	0.069
Level of menstrual flow							
Light or moderate	318	75.2	105	24.8	1		
Heavy	85	82.5	18	17.5	1.42	0.83, 2.45	0.204
Attitude							
Lower attitude	78	70.3	33	29.7	1		
Moderate attitude	117	70.9	48	29.1	1.49	0.90, 2.47	0.123
Higher attitude	208	83.2	42	16.8	3.41	1.99, 5.83	0.001
Perceived behavioral control (PBC)							
Lower PBC	131	67.2	64	32.8	1		
Moderate PBC	117	76.5	36	23.5	1.67	1.03, 2.70	0.038
Higher PBC	155	87.1	23	12.9	3.17	1.83, 5.50	0.001
Subjective norm							
Lower subjective norm	142	66.7	71	33.3	1		
Moderate subjective norm	90	75.6	29	24.4	1.29	0.90, 2.46	0.918
Higher subjective norm	171	88.1	23	11.9	3.40	1.99, 5.83	0.008

*Classified as good if there is no fecal matter, urine, blood, or footprint

**Table 3:** Multivariate analysis with frequency of changing sanitary napkins in school toilets

Variables	Odds ratio	95% CI	*P* value
Observed availability of sanitary bin			
Not available	1		
Available in toilet	2.54	0.76, 8.40	0.128
Available in cubicles	2.19	0.84, 5.74	0.111
Observed toilet bowl cleanliness			
Bad	1		
Good	1.23	0.54, 2.81	0.618
Perceived privacy			
Disagree	1		
Neutral	1.01	0.40, 2.55	0.976
Agree	1.47	0.68, 3.18	0.332
Perceived availability of sanitary bin			
Disagree	1		
Neutral	2.31	0.84, 6.40	0.104
Agree	1.25	0.61, 2.56	0.545
Perceived toilet bowl cleanliness			
Disagree	1		
Neutral	0.64	0.28, 1.48	0.298
Agree	0.62	0.30, 1.29	0.196
Perceived smell			
Disagree	1		
Neutral	1.18	0.51, 2.74	0.693
Agree	0.77	0.42, 1.41	0.393
Perceived availability of hand wash facility			
Disagree	1		
Neutral	1.42	0.53, 3.80	0.479
Agree	1.18	0.57, 2.47	0.655
Age	1.33	0.99, 1.80	0.058
Father’s educational attainment			
Secondary incompletion	1		
Secondary	1.56	0.94, 2.60	0.087
Tertiary	2.09	1.08, 4.02	0.029
Level of menstrual flow			
Light or moderate	1		
Heavy	1.50	0.83, 2.72	0.174
Attitude			
Lower attitude	1		
Moderate attitude	0.88	0.49, 1.60	0.671
Higher attitude	1.23	0.67, 2.27	0.510
Perceived behavioral control (PBC)			
Lower PBC	1		
Moderate PBC	1.57	0.91, 2.69	0.103
Higher PBC	2.29	1.24, 4.25	0.008
Subjective norm			
Lower subjective norm	1		
Moderate subjective norm	1.28	0.74, 2.23	0.375
Higher subjective norm	2.63	1.45, 4.76	0.001

In addition, bivariate analysis showed that all the TPB variables were significantly associated with changing sanitary napkins. Participants with higher attitude score are more than three times more likely to change sanitary napkins in school toilets compared to those who had lower attitude score (COR 3.41; 95% CI 1.99 to 5.83). Participants who reported moderate or higher perceived behavioral control score were significantly more likely to change sanitary napkins in school toilets, compared to those who had lower perceived behavioral control score, respectively (COR 1.67, 95% CI 1.03 to 2.70; COR 3.17; 95% CI 1.83 to 5.50). Participants with higher subjective norm scores were significantly three times more likely to change sanitary napkins compared to those with lower subjective norm scores (COR 3.40; 95% CI 1.99 to 5.83). In multivariate analysis, participants who reported stronger perceived behavioral control and higher subjective norm were significantly more likely to change sanitary napkins, compared to those with lower perceived behavioral control score and subjective norm score (AOR 2.29; 95% CI 1.24 to 4.25; AOR 2.63; 95% CI 1.45 to 4.76).

## Discussion

Unexpectedly, in the present study, the toilet quality variables were not associated with the frequency of changing sanitary napkins in school toilets. However, the absence of the significant association does not necessarily reject the significant effect of the toilet quality on the frequency of changing sanitary napkins. There are at least two possible reasons for the absence of a significant association.

First, the number of schools examined was not sufficient to ensure the variability of toilet quality at school level. For example, there was only one school that lacked sanitary bin (i.e., school 3 as shown in Table 4). Although the association between the observed availability of sanitary bin and frequency of changing sanitary napkins did not reach the statistically significant level, the effect size of the association was substantial. The odds ratio of “available in toilet” to “not available” was 2.54. Therefore, if we included more schools and ensured the variability in the availability of sanitary bin, then we might have found the significant association.

**Table 4:** Results of toilet observation

Variables	Schools					
	1	2	3	4	5	6
Functional flush	✓	✓	✓	✓	✓	✓
Door is intact	✓	✓	✓	✓	✓	
Functional lock		✓				
Toilet interior is not visible from outside	✓		✓			
Availability of sanitary bin	✓	✓		✓	✓	✓
Floor has no litters		✓		✓		
Floor is not wet				✓		
Wall is clean and no scribbles			✓	✓		
Toilet seat and bowl are clean	✓	✓		✓		
Window is available	✓	✓	✓	✓	✓	✓
No objectionable smell		✓		✓		
Artificial light is functional	✓	✓	✓	✓	✓	✓
Bathroom sinks are present	✓	✓	✓	✓	✓	✓
Taps are functional	✓		✓		✓	

✓means okay/yes/available

Second, the measurements for toilet quality used in the present study did not cover all the characteristics of toilet quality, especially the availability of clean water for washing the vagina. Because Ellis reported that one of the MHM challenges encountered by female students in some areas of Philippines was access to water for flushing and washing, there is a possibility that the clean water for washing bodies has the significant effect on changing sanitary napkins in our study site.

Even though a statistically significant association was not found between toilet quality and the frequency of changing sanitary napkins in school, toilet quality should still be given due importance. During the observation survey, half of school toilets did not have functional taps for washing (Table 4). Weaver et al. reported that students from schools with little or no access to hand wash stations were almost twice as likely to report diarrhea episodes (adjusted relative risk 1.95; 95% confidence interval 0.98 to 3.49), compared to students at schools with the highest number of hand wash stations [28]. Toilet quality should therefore be improved regardless of the lack of association.

The present study also showed that 23% of the students did not change sanitary napkins while in school. According to House et al., changing menstrual materials every 2 to 6 h or more frequently is recommended [6]. Although the Department of Education in the Philippines has already included self-management of menstruation in the curriculum for primary schools [29], it would be better for the government to consider complementary intervention to translate knowledge into practice, apart from the existing curriculum.

The present study showed that subjective norm and perceived behavioral control had associations with frequency of changing sanitary napkins. Therefore, there is a possibility that a TPB-based intervention would improve the menstrual hygiene behavior through improved subjective norm and perceived behavior control, in the study site.

Several studies demonstrated the effectiveness of TPB-based intervention in improving some health behaviors. For example, holding sessions with parents, teachers, and role models who shared their successful experiences, and providing posters of positive role models [30, 31]. Those kinds of interventions can be recommended aside from the current school curriculum.

The affordability of sanitary napkins in the study schools should be improved. In the present study, father’s education was significantly associated with changing sanitary napkins (AOR 2.09; 95% CI 1.08 to 4.02). This finding suggests that there might be financial barriers in buying sanitary napkins. The Department of Education in the Philippines has already requested schools to provide stores where students can buy sanitary napkins [32]. This will improve the accessibility of sanitary napkins in the schools, but its affordability should be ensured. Acceptability of the sanitary napkins in terms of quality might be another factor in its utilization.

There are three major limitations in the present study. First, our study was not able to exactly capture the socio-economic status of the respondents that reflects the economic ability to buy sanitary napkins. A more direct method that asks household income or household expenditure might be better to capture the socio-economic status. Second, the present study did not consider the quality of sanitary napkins which can impact the frequency of changing sanitary napkins. Lastly, responses of 526 out of the target 600 participants (considering 30% non-response) were analyzed. Although the target sample size without considering non-response was 450, conclusions would have changed if all of the 850 respondents participated in the study.

In conclusion, there was no significant association between the toilet quality and frequency of changing sanitary napkins. However, it does not mean that the cause-effect relationship was rejected because the variability of toilet characteristics among schools was not enough and the measurements used in the present study were not comprehensive. Future studies should include more schools and also perform comprehensive measurements. Although the present study did not demonstrate the association between toilet quality and frequency of changing sanitary napkins, it demonstrated the significant association between perceived behavioral control and frequency of changing sanitary napkins, and subjective norm and frequency of changing sanitary napkins. An intervention which is grounded from these TPB constructs to improve students’ perceptions can be recommended for promoting better menstrual hygiene management. The present study also demonstrated the significant association between father’s education and changing sanitary napkins.
